# Digital food environment during the coronavirus disease 2019 (COVID-19) pandemic in Brazil: an analysis of food advertising in an online food delivery platform

**DOI:** 10.1017/S0007114520004560

**Published:** 2020-11-19

**Authors:** Paula Martins Horta, Juliana de Paula Matos, Larissa Loures Mendes

**Affiliations:** Departamento de Nutrição, Universidade Federal de Minas Gerais, Belo Horizonte, Minas Gerais, Brasil

**Keywords:** COVID-19, Digital media, Food environment, Food marketing, Brazil, app, application, COVID-19, coronavirus disease 2019, OFD, online food delivery

## Abstract

Online food delivery (OFD) platforms guarantee access to food during the coronavirus disease 2019 (COVID-19) pandemic when commercial food establishments are closed and access to food retail is controlled. The present study aimed to describe the advertisements published in an OFD platform in Brazilian capitals, during the 13th and 14th weeks of the pandemic. Data collection occurred on 1 d of the week and 1 d of the weekend and during lunch and dinner time. A random sample of 25 % of the advertisements (*n* 1754) published in this period was classified in accordance with the presence of food groups and to the use of marketing strategies. Sandwiches, ultra-processed beverages, traditional meals or pasta were the most common food groups shown in the advertisements. Free delivery prevailed in advertisements of ice cream, candies or salty packages snacks and pizza (*P* < 0·01). Combos were more frequently shown in the advertising of natural juices or smoothies, ultra-processed beverages, sandwiches and pizzas (*P* < 0·01). Messages about healthiness were more seen among natural juices or smoothies, vegetables and traditional meals and pasta advertisements (*P* < 0·01) and less seen in sandwiches (*P* = 0·02) and pizza advertisements (*P* < 0·01). Economy messages were rare in advertisements of traditional meals or pasta (*P* < 0·01) and more common in ultra-processed beverages (*P* = 0·03) and ice cream, candies or salty packages snacks (*P* < 0·01) advertisements. The OFD platform promoted unhealthy eating during the COVID-19 pandemic in Brazil due to the expressive presence of unhealthy foods advertising.

In December 2019, several cases of pneumonia appeared in the city of Wuhan, China. Analysis of the genetic material isolated from the virus showed a new *β* coronavirus, the severe acute respiratory syndrome coronavirus 2 (SARS-CoV-2). The disease is called coronavirus disease 2019 (COVID-19) and quickly spread across Chinese territory and the whole world. On 30 January 2020, the WHO declared the disease as a global public health emergency and, on 11 March 2020, as a pandemic with serious health, economic and social impacts^(1,2)^.

In Brazil, the first case of the COVID-19 was confirmed on 29 February 2020. Measures of social distancing are being applied in the country to control the spread of the virus, which involve closing food commercial establishments and controlling access to food retail^(3,4)^.

Consequently, many of the food commercial establishments migrated to take-out and delivery services^(4–6)^ and the use of online food delivery platforms increased in Brazil: during the lockdown, the use of these applications (apps) grew up by 9 % on weekdays and 10 % on weekends^(7)^. However, food services migration to the digital environment and the greater use of these apps by consumers can negatively impact on population’s health since studies prior to the pandemic have characterised the digital food environment as obesogenic by high offer of unhealthy foods^(8,9)^.

Online food delivery platforms also use intensive marketing strategies^(8,10)^, such as photos, discounts, free delivery and combos (a combination of food items and or drinks offered at a discount), mostly directed at unhealthy meals^(8)^. These strategies, especially those that confer some financial benefit to the consumer, can play a prominent role during the COVID-19 pandemic, due to the greater socio-economic vulnerability of the consumer^(11,12)^. In Brazil, during the social distancing period, the pandemic costs are estimated at R$ 20 billion per week^(13)^.

Digital food environment covers social media and the digital marketing^(10)^ and changes in this environment can impact on the population health. Thus, understanding the implications of the COVID-19 pandemic under the digital food environment in Brazil can direct policies and actions that promote healthy eating practices during and after this health crisis^(14)^.

The present study aims to describe the advertisements published in an online food delivery platform in the twenty-seven Brazilian capitals, during the COVID-19 pandemic.

## Methodology

This is an analytic study that investigated food advertising in an online food delivery platform in the twenty-seven Brazilian capitals between the 13th and 14th weeks of the COVID-19 pandemic.

The platform under study was created in Brazil in 2011 and is currently the biggest food tech company in Latin America, being present in Brazil, Mexico and Colombia. In Brazil, at the time of the present study, this platform was the only that served all the Brazilian capitals. In 2019, the platform delivered an average of 13 million orders per month and this number reached 39 million during March 2020, when more than 1·5 million downloads of the app were registered. Inside this platform, consumers can access the menus of the registered restaurants and order ready-to-eat meals. Their orders are forwarded to these food outlets and once ready, meals are delivered to customers by couriers working. Payment method includes meal vouchers or debit/credit cards. Food advertising can be shown on the home page of the app aiming to access all consumers that use the app or inside restaurant menus aiming to access the specific public that is interested in a restaurant.

In our study, all the advertisements (*n* 7005) available on the home page of the app were recorded. We did not include the advertisements made available only inside a restaurant menu.

Data collection was carried out on two non-consecutive days, 1 d of the week and 1 d of the weekend and comprised lunch (11.00–13.00 hours) and dinner time (18.00–21.00 hours). These meals were chosen because they represent, in addition with breakfast (03.00–10.00 hours), the main meals in terms of daily total energy intake contribution in Brazil^(15)^.

A sample of 25 % of the advertisements (*n* 1754) was selected by a randomisation process stratified by the day of the week, the mealtime and the city. Therefore, the selected sample represents the universe of food advertising inside the food delivery app home page according to the Brazilian’s capitals, day of the week and mealtime.

Foods announced in advertisements were classified into the following food groups: water; natural juices and smoothies; vegetables; fruits; traditional meals (dishes predominantly made with unprocessed and minimally processed foods very typical in Brazil) and pasta; ultra-processed beverages; ice cream and candies and salty packaged snacks; sandwiches; savoury snacks; pizzas (Table 1). These food groups were defined based on a previous research that characterised food availability and advertising inside online food delivery platforms in Brazil^(8)^ and are aligned to the NOVA food classification system^(16,17)^ and the Brazilian Dietary Guidelines^(18)^.

**Table 1.** Description of eating markers

Markers	Description
Water	Natural water bottled in traditional and carbonated versions
Natural juices or smoothies	Fruit or vegetable juices and smoothies. Example: orange juice; cabbage, pineapple and ginger juice
Vegetables	Dishes made predominantly from vegetables. Examples: mix of salads, vegetable broth
Fruits	Dishes made predominantly of fruits. Example: fruit salad, apple, banana
Traditional meals or pasta	Dishes predominantly made with unprocessed and minimally processed foods, pasta and international cuisine dishes (except oriental). Examples: rice, beans, meat and vegetables; lasagna and paella
Ultra-processed beverages	Soft drinks, ultra-processed juices, energy drinks, tonic water and flavoured water
Ice cream and candies or salty packaged snacks	Ice cream, popsicles, candies, chewing gum, sweets and chocolates and salty packaged snacks such as chips
Sandwiches	Dishes based on bread and ultra-processed ingredients. Examples: hamburger, hot dogs
Savoury snacks	Fried and baked savoury snacks. Example: croquette
Pizzas	Pizzas made predominantly with ultra-processed ingredients. Example: ham pizzas

The marketing strategies investigated in each advertisement were free delivery, discounts, use of photos, combos (a combination of food items and or drinks offered at a discount) and messages of healthiness, economy and tasty and pleasure.

Double coding of all advertisements was run in an Excel spreadsheet by two independent assessments with eight researchers completing this work. The coding was checked for agreement, and all divergences were solved by a researcher not involved in data collection.

Data analysis was performed using Stata software (version 14.0). Advertisements were rated for the presence of food groups and marketing strategies participation and were described on the weekdays (Monday, Tuesday, Wednesday, Thursday and Friday) and on the weekend (Saturday and Sunday) as well as at lunch and dinner and according to the regions of the country (North/Northeast/Midwest and South/Southeast). The differences were tested by applying Pearson’s *χ* ^2^ test at a 5 % significance level (*P* < 0·05). This test was also performed to compare the participation of marketing strategies in the presence of food groups advertising.

## Results

Advertisements were published equally between the days of the week (weekday: 51·4 %; weekend: 48·6 %) and were concentrated on dinner (58·3 %; 41·7 % at lunch).

Sandwiches, ultra-processed beverages, traditional meals or pasta and pizzas were the most common food groups shown in the advertisements. The other food groups were present in less than 5 % of the sample. Vegetables and traditional meals or pasta were more advertised at lunch (*P* < 0·01). The latter were also more exhibited on the weekday advertising (*P* < 0·01). In contrast, ultra-processed beverages, sandwiches and pizzas were more advertised at dinner (*P* < 0·01). Pizzas were also more frequent in advertisements for the Southeast and South regions (*P* = 0·04) (Table 2).

**Table 2.** Participation of food groups on advertisements in an online food delivery platform in accordance with the day of the week, mealtime, and region of the country

Food groups	Total sample (%)	Day of the week (%)			Mealtime (%)			Region of the country (%)		
		Weekday	Weekend	*P*	Lunch	Dinner	*P*	North/Northeast/Midwest	Southeast/South	*P*
Water	0·1	0·0	0·1	0·30	0·0	0·1	0·40	0·1	0·0	0·45
Natural juices or smoothies	0·8	0·8	0·8	0·88	0·9	0·8	0·70	1·1	0·5	0·19
Vegetables	2·5	2·9	2·0	0·23	3·8	1·5	<0·01	2·1	3·1	0·16
Fruits	1·3	1·6	0·9	0·25	1·5	1·1	0·43	1·5	0·8	0·18
Traditional meals or pasta	17·8	21·1	14·4	<0·01	31·3	8·2	<0·01	25·6	25·0	0·79
Ultra-processed beverages	20·6	21·5	19·7	0·36	16·0	23·9	<0·01	21·8	18·6	0·10
Ice cream, candies or salty packaged snacks	4·2	3·6	4·8	0·19	4·5	3·9	0·54	4·8	2·9	0·06
Sandwiches	21·3	20·2	22·5	0·23	13·8	26·7	<0·01	20·9	22·1	0·59
Savoury snacks	4·3	4·0	4·7	0·47	3·6	4·9	0·17	3·7	5·5	0·07
Pizzas	16·7	15·3	18·1	0·12	4·9	25·1	<0·01	15·3	19·0	0·04

Almost all advertisements used photos or discounts and about half of the sample used free delivery. Combos (*P* = 0·01) and photos (*P* = 0·03) were more common on the weekdays advertising, while tasty and pleasure messages prevailed at the weekend (*P* < 0·01). Free delivery (*P* = 0·02), combo (*P* < 0·01) and messages of economy (*P* < 0·01) were more common on advertisements spread at dinner, while the use of photos and healthiness messages were more frequent at lunch advertising (*P* < 0·01). In addition, free delivery (*P* < 0·01), discount (*P* < 0·01) and economy messages (*P* = 0·02) were more frequent in the Southeast/South advertising (Table 3).

**Table 3.** Participation of marketing strategies on advertisements in an online food delivery platform in accordance with the day of the week, mealtime and region of the country

Marketing characteristics	Total sample (%)	Day of the week (%)			Mealtime (%)			Region of the country (%)		
		Weekday	Weekend	*P*	Lunch	Dinner	*P*	North/Northeast/Midwest	Southeast/South	*P*
Free delivery	45·7	46·3	44·9	0·56	42·4	48·0	0·02	39·5	56·5	<0·01
Discount	91·7	91·0	92·5	0·26	90·9	92·3	0·34	89·5	95·8	<0·01
Combo	29·3	32·1	26·4	0·01	23·6	33·4	<0·01	29·7	28·6	0·63
Photo	94·0	95·2	92·7	0·03	96·2	92·5	<0·01	93·8	94·3	0·67
Message of healthiness	2·9	3·2	2·6	0·43	4·9	1·5	<0·01	2·6	3·5	0·30
Message of economy	15·7	15·5	15·9	0·85	11·6	18·6	<0·01	14·1	18·4	0·02
Message of tasty and pleasure	12·2	8·8	15·9	<0·01	11·5	12·7	0·43	11·5	13·5	0·20

Finally, free delivery prevailed in advertisements of ice cream, candies or salty packages snacks and pizza (*P* < 0·01) and was less common among advertisements about sandwiches (*P* = 0·02). Combos were more frequently shown in the advertising of natural juices or smoothies, ultra-processed beverages, sandwiches and pizzas (*P* < 0·01). However, combos were less commonly used when the advertisement was about traditional meals or pasta (*P* < 0·01). Photos were more commonly used in ice cream, candies or salty packages snacks (*P* = 0·03) advertisements, while this strategy was less common in ultra-processed beverages advertisements (*P* = 0·04). Messages about healthiness were more seen among natural juices or smoothies, vegetables and traditional meals and pasta advertisements (*P* < 0·01). In contrast, these messages were rarer in sandwiches (*P* = 0·02) and pizza advertisements (*P* < 0·01). Economy messages were less seen in advertisements of traditional meals or pasta (*P* < 0·01) and more seen in ultra-processed beverages (*P* = 0·03) and ice cream, candies or salty packages snacks (*P* < 0·01) advertisements. Tasty and pleasure messages were more present in sandwiches and savoury snacks advertisements (*P* < 0·01) while less common in ultra-processed beverages (*P* = 0·03) and pizzas (*P* < 0·01) ones (Table 4).

**Table 4.** Participation of marketing strategies on advertisements in an online food delivery platform in accordance with the food groups

Food groups – frequency (%)	Marketing strategy				Marketing messages		
	Free delivery	Discount	Combo	Photo	Healthiness	Economy	Tasty and pleasure
Water							
No	45·6	91·7	29·3	94·0	2·9	15·7	12·2
Yes	100·0	100·0	100·0	100·0	0·0	0·0	0·0
*P*	0·28	0·76	0·12	0·80	0·86	0·67	0·71
Natural juices and smoothies							
No	45·8	91·8	28·8	94·1	2·8	15·7	12·1
Yes	26·7	80·0	86·7	86·7	20·0	20·0	26·7
*P*	0·14	0·09	<0·01	0·23	<0·01	0·64	0·09
Vegetables							
No	45·6	91·9	29·6	94·0	2·3	15·8	12·3
Yes	48·8	86·1	18·6	93·0	25·6	9·3	6·7
*P*	0·67	0·17	0·12	0·78	<0·01	0·24	0·29
Fruits							
No	59·1	91·7	29·5	94·0	2·8	15·7	12·2
Yes	45·5	95·5	18·2	95·5	9·1	18·2	9·1
*P*	0·20	0·52	0·25	0·77	0·08	0·75	0·65
Traditional meals or pasta							
No	46·1	91·7	32·4	93·9	2·2	17·8	12·9
Yes	43·8	92·0	15·0	94·6	6·4	5·8	8·9
*P*	0·46	0·84	<0·01	0·65	<0·01	<0·01	0·05
Ultra-processed beverages							
No	45·9	92·1	11·6	94·0	3·3	14·7	13·1
Yes	44·5	90·3	97·2	91·7	1·4	19·3	8·8
*P*	0·61	0·28	<0·01	0·04	0·05	0·03	0·03
Ice cream, candies or salty packaged snacks							
No	44·9	91·7	29·7	93·8	3·0	15·2	11·9
Yes	63·0	93·2	20·6	100·0	1·4	27·4	17·8
*P*	<0·01	0·65	0·09	0·03	0·42	<0·01	0·14
Sandwiches							
No	47·1	91·5	24·4	94·1	3·4	16·5	10·7
Yes	40·4	92·5	47·3	93·9	1·1	12·8	17·7
*P*	0·02	0·54	<0·01	0·88	0·02	0·09	<0·01
Savoury snacks							
No	45·8	91·6	28·9	93·9	3·0	15·6	11·7
Yes	42·1	94·7	38·2	96·1	1·3	18·4	22·4
*P*	0·52	0·33	0·08	0·44	0·39	0·50	<0·01
Pizzas							
No	44·2	91·9	25·0	94·3	3·4	12·2	13·2
Yes	53·1	91·1	50·7	92·8	0·3	33·2	7·2
*P*	<0·01	0·67	<0·01	0·34	<0·01	<0·01	<0·01

## Discussion

The digital food environment represented by an online food delivery platform proved to be obesogenic in Brazil due to the expressive presence of unhealthy foods advertising during the COVID-19 pandemic. Characteristics related to the day of the week, mealtime and region of the country differentiated the advertisements.

The study described for the first time the profile of advertisements in a food delivery app in all Brazilian capitals in a moment when food commercial establishments were closed and the digital food environment represented an important way of acquiring ready-to-eat meals^(4)^. The study showed that unhealthy foods prevailed in the advertisements, mainly due to the presence of ultra-processed beverages and sandwiches, while the healthy foods, mainly represented by traditional meals and pasta, were advertised less frequently. Marketing strategies were more directed to advertisements containing unhealthy foods and prevailed the use of photos, discounts and free delivery.

In other words, when the digital food environment becomes one of the main means of acquiring ready-to-eat meals in the world^(5,6,19)^, advertisements promoted by restaurants registered in a food delivery app in Brazil were mostly focused on unhealthy meals and used marketing strategies in order to persuade the consumer to purchase these products.

Study conducted in 2019 described the use of photos and discounts and the menus of the restaurant registered in two online food delivery platforms in a Brazilian capital: ultra-processed beverages were present in 78·5 % of the restaurant menus, while water and natural juices and smoothies appeared in 49 and 27 % of the menus, respectively. Sandwiches, pizzas and fried snacks were options in 39, 13·8 and 16·6 % of the menus, while traditional meals were checked in 20·4 % of restaurant menus and vegetables in 16·9 %. The use of photos and discounts were more frequent in menus that offered meals containing unhealthy food groups compared with meals containing unprocessed and minimally processed foods^(8)^.

The frequent use of marketing strategies in food delivery platforms aims to persuade the consumers to buy inside the apps at the same time they are having a great experience of shopping. The literature has pointed out some potential drivers of online food delivery service: convenience, usage usefulness and hedonic motivations^(20–22)^. The marketing strategies used by the online food delivery platform under study aim to confer these characteristics to the digital environment.

The intense desire for convenience food is a result of rising household incomes, urbanisation and a reduction in the time available for activities such as cooking. More generally, reasons given for consuming meals prepared away from home include the desire for fast and filling meals, the cost and effort of cooking at home and a lack of time. Also, the usage usefulness of the food delivery apps is related to the costumers’ interest on technology that can provide saving time and effort. Thus, the website must be user friendly and be able to process the customer’s request as quickly as possible^(20–22)^. Combos strategy, for example, can help individuals chose a meal in an extremely fast way. Having certain discounts or promotions may also attract price-sensitive consumers, as they are likely to choose the channel which provides them the best value for money^(20–22)^. However, the time-saving factor is not the only motivation for using online food delivery platforms. Shopping motivations can also come from values and pleasure that consumer seeks from shopping^(20–22)^. Using photos and messages of healthiness or tasty and pleasure can enhance the hedonic experience of the consumer when shopping online.

In our study, the majority of the advertisements that contained marketing strategies, except for healthiness messages, was mainly directed to unhealthy food groups which is especially undesirable during the COVID-19 pandemic, as several studies have shown associations between the increased risk of mortality from this disease in obese patients and those with diabetes and hypertension^(23,24)^. Thus, in addition to the problem of stimulating an unhealthy diet for a population that is currently 55·4 % overweight and 20·3 % obese^(25)^, increasing body weight in this epidemiological moment is linked to a worse prognosis for the COVID-19^(23,24)^.

The study has also pointed out differences in the profile of advertisements and marketing strategies use according to the mealtime, day of the week and region of the country. This differentiation is in line with the dietary pattern present in the country and the operational logic of commercial food establishments. For example, during lunch and during the week we noticed a greater promotional offer of traditional meals and vegetables. In Brazil, the consumption of vegetables at lunchtime is twice the amount consumed at dinner^(26)^ and, the Brazilian, in comparison with other populations, preserves the habit of having traditional meals, as well as the consumption of rice and beans^(27–29)^. In addition, self-service or kilo food restaurants operate during lunch hours, while pizzerias and hamburgers shops operate more prevalently at night and on weekends.

A greater participation of free delivery, combos and economy messages was also more noted for the dinner, when the presence of unhealthy foods advertising is higher. In contrast, the greatest use of photos and healthiness messages was found during the lunch period, which is the period of greatest supply of healthy food groups. Additionally, the tasty and pleasure messages were more common on the weekend period. Considering the regionality, restaurants of the North, Northeast and Midwest regions used less marketing strategies than the restaurants of the Southeast and South of Brazil. The profile of food acquisition in the North, Northeast and Midwest is characterised by a greater participation of unprocessed or minimally processed foods and a lower share of ultra-processed foods^(27)^, which may explain the lesser incentive to consume unhealthy food groups in these regions.

Finally, it is worth highlighting the role that food delivery apps can exert for guaranteeing access to food during the COVID-19 pandemic. The predominance of using the digital media for acquiring food will remain after the pandemic as a safety measure to reduce all types of transmission of viral diseases^(14)^. In spite of this, the relative newness of the online food delivery platforms put them as a new route to the market as they do not fit into the categories of foodservice outlet, food retail outlet or manufacturer, which is a factor in its absence from public health policies^(22)^.

In this regard, food delivery apps can be improved, for example, by including the list of ingredients and the nutritional information of the announced meals and reformulating portion size^(22)^. In addition, these platforms need to be regulated by specific legislation in line with the known impact of food marketing on the health profile of the population, in order to not become another marketing space for unhealthy foods^(14,30)^. The present study contributes to this discussion by providing a description of the food advertising in an online food delivery platform in Brazil during the COVID-19 pandemic and points out possibilities for regulation so that this digital food environment is healthier for the population during and after this sanitary context.

Despite being unprecedented and relevant to the current health context in Brazil, the study has limitations that need to be highlighted. First, data collection involved all advertisements made by restaurants in the country’s twenty-seven capitals between the 13th and 14th weeks of the COVID-19 pandemic. Therefore, data collection occurred in an initial moment of the pandemic and by the absence of previous data describing the profile of advertisements in food delivery apps, it is not possible to state that the data described here represent a different scenario than the one before the installation of the pandemic. Despite this, data collection occurred at a time when the food delivery apps use had boosted in Brazil^(7)^.

Furthermore, due to the fact that the study is not longitudinal and, therefore, did not describe all the advertisements published throughout the COVID-19 pandemic in Brazil, it is not possible to state that the scenario described represents the profile of advertisements throughout the installation of the pandemic in the country. The present group of researchers is addressing this issue by studying the profile of food advertisements published in delivery apps in a Brazilian capital during the months that followed the beginning of the pandemic.

Still, the study focused on the description of advertisements spread in a food delivery app, which does not refer to the totality of what is offered by the restaurants registered on this platform. Furthermore, other food delivery companies also operate online and characterise the digital food delivery environment in Brazil. In this way, the results of this investigation portray exclusively the advertising strategies that restaurants registered in a food delivery app used to boost sales during the pandemic of the COVID-19 in Brazil.

## Ethical statement

The present study does not involve human participants.

## References

[1] Binns C , Low WY & Kyung LM (2020) The COVID-19 pandemic: public health and epidemiology. Asia Pac J Public Health 32, 140–144. 32429675 10.1177/1010539520929223 PMC7240312

[2] Zhou G , Chen S & Chen Z (2020) Back to the Spring of 2020: facts and hope of COVID-19 outbreak. Front Med 14, 113–119. 32172487 10.1007/s11684-020-0758-9 PMC7089213

[3] Ministério da Saúde, Brasil (2020) Diretrizes para diagnóstico e tratamento da COVID-19 (Guidelines for the Diagnosis and Treatment of COVID-19). Brasília: Ministério da Saúde.

[4] Oliveira TC , Abranches MV & Lana RM (2020) Food (in)security in the context of the SARS-Cov-2 pandemic. Cad Saude Publica 36, e00055220. 32267383 10.1590/0102-311X00055220

[5] Galanakis CM (2020) The food systems in the Era of the Coronavirus (COVID-19) pandemic crisis. Foods 9, 523. 10.3390/foods9040523 PMC7230343 32331259

[6] Petetin L (2020) The COVID-19 crisis: an opportunity to integrate food democracy into post-pandemic food systems. Eut J Risk Regul 11, 326–336.

[7] Kantar Ibope Media (2020) Kantar aponta as principais transformações e as tendências de comportamento do consumidor pós-quarentena (Kantar points out the main transformations and trends in post-quarantine consumer behaviour). https://www.kantaribopemedia.com/kantar-aponta-as-principais-transformacoes-e-as-tendencias-de-comportamento-do-consumidor-pos-quarentena/ (accessed September 2020).

[8] Horta PM , Souza JPM , Rocha LL , et al. (2020) Digital food environment of a Brazilian metropolis: food availability and marketing strategies used by delivery apps. Public Health Nutr (epublication ahead of print version 9 September 2020). 10.1017/S1368980020003171 PMC10195591 32900419

[9] Poelman MP , Thornton L & Zenk SN (2020) A cross-sectional comparison of meal-delivery options in three international cities. Eur J Clin Nutr 74, 1465–1473. 32332863 10.1038/s41430-020-0630-7

[10] Granheim SI , Opheim E , Terragni L , et al. (2020) Mapping the digital food environment: a scoping review protocol. BMJ Open 10, e036241. 10.1136/bmjopen-2019-036241 PMC7204828 32327482

[11] Abrams EM & Szefler SJ (2020) COVID-19 and the impact of social determinants of health. Lancet Respir Med 8, 659–661. 32437646 10.1016/S2213-2600(20)30234-4 PMC7234789

[12] Nicola M , Alsafi Z , Sohrabi C , et al. (2020) The socio-economic implications of the coronavirus and COVID-19 pandemic: a review. Int J Surg 78, 185–193. 32305533 10.1016/j.ijsu.2020.04.018 PMC7162753

[13] Ministério da Economia, Brasil (2020) Nota informativa; impactos econômicos da COVID-19 (Informative note; economic impacts of COVID-19). http://www.fazenda.gov.br/centrais-de-conteudos/publicacoes/conjuntura-economica/estudos-economicos/2019/nota-impactos-economicos-da-covid-19.pdf (acessed March 2020).

[14] Bakallis S , Valdramidis V , Argyropoulos D , et al. (2020) Perspectives from CO+RE: How COVID-19 changed our food systems and food security paradigms. Cur Res Food Sci 3, 166–172. 10.1016/j.crfs.2020.05.003 PMC7265867 32908972

[15] Gombi-Vaca MF , Sichieri R & Verly E Jr (2016) Caloric compensation for sugar-sweetened beverages in meals: a population-based study on Brazil. Appetite 98, 67–73. 26708263 10.1016/j.appet.2015.12.014

[16] Monteiro CA , Levy RB , Claro RM , et al. (2010) A new classification of foods based on the extent and purpose of their processing. Cad Saude Publica 26, 2039–2049. 21180977 10.1590/s0102-311x2010001100005

[17] Monteiro CA , Cannon G , Levy R , et al. (2016) NOVA: the star shines bright. Food classification. World Nutr 7, 28–38.

[18] Ministério da Saúde, Brasil (2014) Guia alimentar para a população brasileira (Food Guide for the Brazilian Population). Brasília: Ministério da Saúde.

[19] Rodrigues MB , Matos JP & Horta PM (2020) The COVID-19 pandemic and its implication for the food information environment in Brazil. Public Health Nutr (Epublication ahead of print version 23 November 2020). 10.1017/S1368980020004747 PMC7737163 33222707

[20] Yeo V , Goh S & Rezaei S (2017) Consumer experiences, attitude and behavioural intention toward online food delivery (OFD) services. J Retail Consum Serv 35, 150–162.

[21] Maimaiti M , Zhao X , Jia M , et al. (2018) How we eat determines what we become: opportunities and challenges brought by food delivery industry in a changing world in China. Eur J Clin Nutr 72, 1282–1286. 30185849 10.1038/s41430-018-0191-1

[22] Bates S , Reeve B & Trevena H (2020) A narrative review of online food delivery in Australia: challenges and opportunity for public health nutrition policy. Public Health Nutr (epublication ahead of print version 9 June 2020). 10.1017/S1368980020000701 PMC7613985 32515719

[23] Dietz W & Santos-Burgoa C (2020) Obesity and its implication for COVID-19 mortality. Obesity 28, 1005. 32237206 10.1002/oby.22818

[24] Kassir R (2020) Risk of COVID-19 for patients with obesity. Obes Rev 21, e13034. 32281287 10.1111/obr.13034 PMC7235532

[25] Ministério da Saúde, Brasil (2020) Vigilância de Fatores de Risco e Proteção para Doenças Crônicas por Inquérito Telefônico: estimativas sobre frequência e distribuição sociodemográfica de fatores de risco e proteção para doenças crônicas nas capitais dos 26 estados brasileiros e no Distrito Federal em 2019 (Surveillance of Risk and Protection Factors for Chronic Diseases by Telephone Survey: Estimates on the Frequency and Sociodemographic Distribution of Risk and Protective Factors for Chronic Diseases in the Capitals of the 26 Brazilian States and the Federal District in 2019). Brasília: Ministério da Saúde.

[26] Canella DS , Louzada MLC , Claro RM , et al. (2018) Consumo de hortaliças e sua relação com os alimentos ultraprocessados (Vegetable consumption and its relationship with ultra-processed foods). Rev Saude Publica 52, 50. 29791530 10.11606/S1518-8787.2018052000111 PMC5953550

[27] Instituto Brasileiro de Geografia e Estatística, Brasil (2020) Pesquisa de Orçamentos Familiares 2017/2018: Avaliação Nutricional da Disponibilidade Domiciliar de Alimentos no Brasil (2017/2018 Household Budget Survey: Nutritional Assessment of Household Food Availability in Brazil). https://biblioteca.ibge.gov.br/visualizacao/livros/liv101670.pdf (accessed March 2020).

[28] Vandevijvere S , Jaacks LM , Monteiro CA , et al. (2019) Global trends in ultra-processed food and drink product sales and their association with adult body mass index trajectories. Obesity Rev 20, 10–19. 10.1111/obr.12860 31099480

[29] Louzada MLC , Martins APB , Canella DS , et al. (2015) Ultra-processed foods and the nutritional dietary profile in Brazil. Rev Saude Publica 49, 38. 26176747 10.1590/S0034-8910.2015049006132 PMC4544452

[30] Buchanan L , Kelly B , Yeatman H , et al. (2018) The effects of digital marketing of unhealthy commodities on young people: a systematic review. Nutrients 10, 14. 10.3390/nu10020148 PMC5852724 29382140

